# High Throughput Kinomic Profiling of Human Clear Cell Renal Cell Carcinoma Identifies Kinase Activity Dependent Molecular Subtypes

**DOI:** 10.1371/journal.pone.0139267

**Published:** 2015-09-25

**Authors:** Joshua C. Anderson, Christopher D. Willey, Amitkumar Mehta, Karim Welaya, Dongquan Chen, Christine W. Duarte, Pooja Ghatalia, Waleed Arafat, Ankit Madan, Sunil Sudarshan, Gurudatta Naik, William E. Grizzle, Toni K. Choueiri, Guru Sonpavde

**Affiliations:** 1 Department of Radiation Oncology, University of Alabama at Birmingham, Birmingham, Alabama, United States of America; 2 Division of Hematology and Oncology, University of Alabama at Birmingham, Birmingham, Alabama, United States of America; 3 Division of Preventative Medicine, University of Alabama at Birmingham, Birmingham, Alabama, United States of America; 4 Department of Urology, University of Alabama at Birmingham, Birmingham, Alabama, United States of America; 5 Division of Anatomic Pathology, University of Alabama at Birmingham, Birmingham, Alabama, United States of America; 6 Clinical Oncology Department, University of Alexandria, Alexandria, Egypt; 7 Center for Outcomes Research and Evaluation (CORE), Maine Medical Center Research Institute (MMCRI), Portland, Maine, United States of America; 8 Department of Medical Oncology, Kidney Cancer Center, Dana-Farber Cancer Institute and Harvard Medical School, Boston, Massachusetts, United States of America; Rutgers University, UNITED STATES

## Abstract

Despite the widespread use of kinase-targeted agents in clear cell renal cell carcinoma (CC-RCC), comprehensive kinase activity evaluation (kinomic profiling) of these tumors is lacking. Thus, kinomic profiling of CC-RCC may assist in devising a classification system associated with clinical outcomes, and help identify potential therapeutic targets. Fresh frozen CC-RCC tumor lysates from 41 clinically annotated patients who had localized disease at diagnosis were kinomically profiled using the PamStation®12 high-content phospho-peptide substrate microarray system (PamGene International). Twelve of these patients also had matched normal kidneys available that were also profiled. Unsupervised hierarchical clustering and supervised comparisons based on tumor vs. normal kidney and clinical outcome (tumor recurrence) were performed and coupled with advanced network modeling and upstream kinase prediction methods. Unsupervised clustering analysis of localized CC-RCC tumors identified 3 major kinomic groups associated with inflammation (A), translation initiation (B), and immune response and cell adhesions (C) processes. Potential driver kinases implicated include PFTAIRE (PFTK1), PKG1, and SRC, which were identified in groups A, B, and C, respectively. Of the 9 patients who had tumor recurrence, only one was found in Group B. Supervised analysis showed decreased kinase activity of CDK1 and RSK1-4 substrates in those which progressed compared to others. Twelve tumors with matching normal renal tissue implicated increased PIM’s and MAPKAPK’s in tumors compared to adjacent normal renal tissue. As such, comprehensive kinase profiling of CC-RCC tumors could provide a functional classification strategy for patients with localized disease and identify potential therapeutic targets.

## Introduction

Clear cell (CC)-renal cell carcinoma (RCC) is a chemotherapy and radiation resistant tumor affecting approximately 50,000 patients per year in the United States.[1] Surgical resection of localized disease is used with curative intent, yet a substantial percentage of patients will recur both locally and mostly distantly. Historically, systemic therapy for metastatic disease has been disappointing with limited efficacy when employing immunomodulatory therapy (Interleukin-2 and Interferon-alpha).[2, 3] In the past decade, however, several kinase-directed therapies targeting vascular endothelial growth factor (VEGF) and mammalian target of rapamycin (mTOR) have been approved for CC-RCC while several novel kinase-targeted agents are in various stages of preclinical and clinical development for this disease.

Importantly, large research efforts, including the recently reported genomic molecular characterization of CC-RCC by The Cancer Genome Atlas (TCGA) Research Network, have defined or confirmed that CC-RCC is a disease that can harbor alterations in epigenetic regulation (including dysfunction or loss of Von-Hippel Lindau, or VHL) leading to angiogenesis (including increased signaling via VEGF, platelet derived growth factor (PDGF) and fibroblast growth factor (FGF) receptors), proliferation, metabolic disturbances (such as the “Warburg effect”), chromatin remodeling (mutations in PBAF) and altered kinase signaling (including changes in Phosphatidyl-inositol 3-Kinase [PI3K]-Akt-mTOR).[4–6] Surprisingly, while kinase signaling is heavily implicated in CC-RCC, to date, there have been no published large high-throughput screens of kinase activity in panels of primary CC-RCC tumors. Nevertheless, antibodies and small molecule inhibitors targeting kinase dependent pathways are in clinical use for advanced disease including sorafenib (targeting VEGFR, PDGFR, Raf), sunitinib (targeting VEGFR, PDGFR, c-KIT, CSF1R, FLT3, RET), axitinib (VEGFR1-3, PDGFR, c-KIT), bevacizumab (binding VEGF-A), pazopanib (VEGFR1-3, PDFGR, c-KIT), everolimus and temsirolimus (targeting mTOR).[7, 8] Agents targeting c-Met and FGFR in addition to VEGFRs (cabozantinib and lenvatinib) appear promising and are being developed.[9–11].Indeed, cabozantinib was recently reported to significantly extend progression-free survival following prior VEGF inhibitors in a phase III trial and is likely to be approved for commercial use [12].

These agents have provided incremental benefits, however, selection of the most efficacious agent(s), especially in the face of inherent and acquired resistance as well as potential toxicities necessitates improved methods of patient selection and biomarker development. Although several prognostic and predictive biomarkers have been explored in recent years,[13, 14] the investigation of kinase activity in the setting of CC-RCC has been minimal. Therefore, we have utilized a multiplex peptide substrate microarray platform to evaluate global kinase (“kinome”) activity within clinically annotated primary CC-RCC tissue specimens derived from patients undergoing definitive surgery for localized disease.

## Materials and Methods

### Clear cell renal cell carcinoma patient tumors

Fresh frozen CC-RCC and matched adjacent normal kidney tissue (when available) were provided by the Cooperative Human Tissue Network (CHTN) (http://www.chtn.nci.nih.gov) which is funded by the National Cancer Institute. Other investigators may have received specimens from the same subjects. All divisions of the CHTN operate with the review and approval of their local Institutional Review Board (IRB). The following policies and procedures govern collection of specimens and their distribution to investigators and can be found on the CHTN website: “1) CHTN specimens are derived from material that is removed as part of routine medical care or autopsy specimens collected in accordance with operative state and local law. Residual material not needed for patient care is distributed for research. 2) Every CHTN institution has obtained human subjects assurance from the Office of Human Research Protections, DHHS. The Assurance document provides agreement that the institution will comply with federal human subjects regulations (The "Common Rule;" 45 CFR part 46). 3) Each Division of the CHTN is approved by its local IRB to collect and distribute biospecimens. The IRBs review the procedures in place to ensure adequate protection of human subjects and protection of patient privacy and confidentiality. The approvals are reviewed by the IRB yearly.” “The CHTN has always protected the identity of patients from whom specimens are obtained. However, the HIPAA Privacy Rule imposes new requirements on the use of information associated with the specimens. In order to meet the requirements of the Privacy Rule for providing specimens with associated data, the CHTN implemented a Data Use Agreement. The Data Use Agreement permits (investigators) to receive tissue and associated patient information from any division of the CHTN from which such data is available.” As such, an IRB approved protocol (X120917005) at The University of Alabama at Birmingham (UAB) permitted the study in accordance with the Declaration of Helsinki.

**Fig 1:**
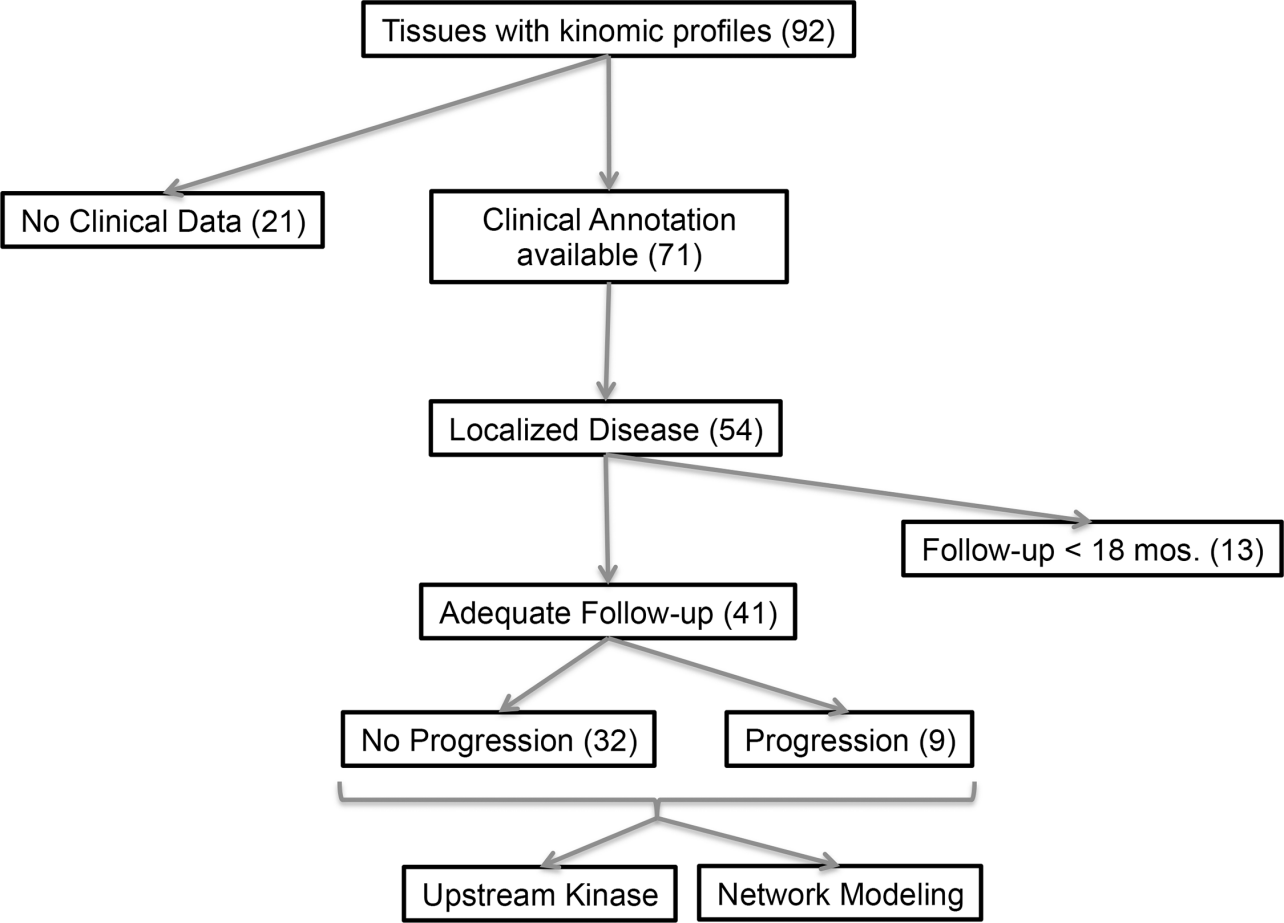
Study Design.

Surgically resected tumor samples were taken from patients, and snap frozen with or without O.C.T.^™^ cryo-embedding media in the gas phase of liquid nitrogen (LN02) according to institutional guidelines,[15] and stored long term in plastic cassettes within computer monitored LN02 tanks. All specimens had independent central pathological assessment for diagnosis confirmation prior to release to the investigator. Patients with localized tumors undergoing surgery with minimum follow-up of 18 months, or documented tumor progression underwent kinomic analysis of fresh frozen CC-RCC tumors (see “Kinomic profiling **”** below). The study design is demonstrated in Fig 1. The characteristics of the patients are listed in Table 1.

**Table 1:** Patient characteristics.

Parameter	Number (%)
N	41
Age, Median	61
Male	29 (70.7)
Female	12 (29.3)
T-Stage	
T1	22 (53.7)
T2	1 (2.4)
T3	17 (41.5)
T4	1 (2.4)
N-Stage	
N0/Nx	38 (92.7)
N1	3 (7.3)
Stage Grouping	
I	19 (46.3)
II	1 (2.4)
III	20 (48.8)
IV	1 (2.4)

### Kinomic profiling

Each of the tissue samples was analyzed, with each tumor lysed independently and analyzed on a separate array. Kinomic profiling of 2–10 μg of protein lysates (lysed in M-Per lysis buffer, Pierce Scientific) containing 1:100 Halt’s protease and phosphatase inhibitors (Pierce cats. 78420, 78415) was conducted on the PamStation® 12 platform, manufactured by PamGene (‘s-Hertogenbosch, Netherlands) within the UAB Kinome Core (www.kinomecore.com) as described previously.[16] After protein quantification (BCA protein determination, Pierce Scientific), total soluble protein lysates were loaded onto the appropriate PamChip® [PTK (tyrosine kinome) or STK (serine/threonine kinome)] in kinase buffer. This platform utilizes a high throughput peptide microarray system analyzing 144 individual tyrosine phosphorylatable peptides, or 144 serine and threonine phosphorylatable peptides imprinted and immobilized in a 3D format to assess kinomic activity in cell or tissue lysates. As such, molecular profiles of each patient’s tumor were measured by kinase activity against 288 phosphorylatable peptides (phosphopeptides) containing a distinct 12–15 amino acid sequence (comprising over 560 phosphorylatable tyrosine, serine, or threonine residues in total). FITC conjugated phospho-specific antibodies (PamGene) are used for visualization during and after lysates are pumped through the array. Capture of peptide phosphorylation signal is via a computer-controlled CCD camera. Kinomic profiling was analyzed using software including Evolve (PamGene) for initial sample and array processing as well as image capture, and BioNavigator (PamGene) for raw data transformation into kinetic (initial velocity) and steady state (postwash) values.

### Data Analysis

Peptide spot intensity (brightness) was captured across multiple exposure times (10, 20, 50,100, 200 ms) and the slope was taken, multiplied by 100, and log2 transformed. This per spot value was used as the ‘signal’. Unsupervised hierarchical clustering of peptide signals for each sample was performed in BioNavigator and is displayed as heatmaps. Specifically, the hierarchical clustering was performed on both the columns and rows using euclidean distance metrics and complete linkage. To test robustness of the clusters, raw data was filtered to remove very low signal strength values and were clustered by columns using the pvclust R script (described here, http://www.sigmath.es.osaka-u.ac.jp/shimo-lab/prog/pvclust/) to generate “*p*-values” for each branch of the dendrogram using bootstrap resampling (both normal bootstrap probability “BP” value, and multiscale approximately unbiased “AU” resampling). AU values of 95 or greater were considered highly supported by the data.[17] The peptide lists within the clusters were then analyzed for upstream kinase prediction using the Kinexus Kinase Predictor (www.phosphonet.ca) with scoring according to the % hits (occurrence divided by the number of residues with kinase information) of a kinase within the top 10 list for each peptide. Network modeling of clusters was also performed by uploading the parent protein Uniprot ID for each peptide to GeneGo MetaCore (portal.genego.com, Thompson Reuters) for Process Mapping. Supervised analysis of the progressors (recurrent tumors) versus non-progressors (disease-free) was also performed using an unpaired student’s t-test to identify significantly altered peptides. In other words, data from samples that were run in singlicate from each tissue specimen were grouped into either progressors or non-progressors and then compared using the statistical test. These peptides were also analyzed using the upstream kinase predictor strategy as above. In addition, 12 CC-RCC tumor specimens were directly compared with matched adjacent normal kidney tissue specimens at the kinome level using a student’s t-test. Significantly altered peptides (p<0.001) were identified and used for upstream kinase prediction and MetaCore analysis as above. Additional analyses of kinomic and clinical data included Fisher’s Exact Testing of progression status, clinical stage and kinomic groups.

## Results

### Patient characteristics

Of the 96 available fresh frozen CC-RCC tumor samples in the CHTN, 92 specimens demonstrated acceptable quality control criteria for kinomic analysis. Of these, 71 tumors had clinical annotation available. When we further restricted the samples to those with localized disease at diagnosis (54) and adequate follow-up (>18 months for the non-progressed group), there were 41 evaluable patients (Study design shown in Fig 1). The clinical-pathological status for these patients is shown in Table 1 and a detailed description for each patient is shown in S1 Table. These patients had a median age of 61 and pathologic stage of I, II, III and IV in 19, 1, 20 and 1 patients, respectively. Of the 41 evaluable CC-RCC tumor specimens that were kinomically profiled, 12 tumors had matching adjacent normal kidney tissue available for kinomic analysis.

### Kinomic profiling of localized CC-RCC tumor samples reveals three distinct kinome clusters

**Fig 2:**
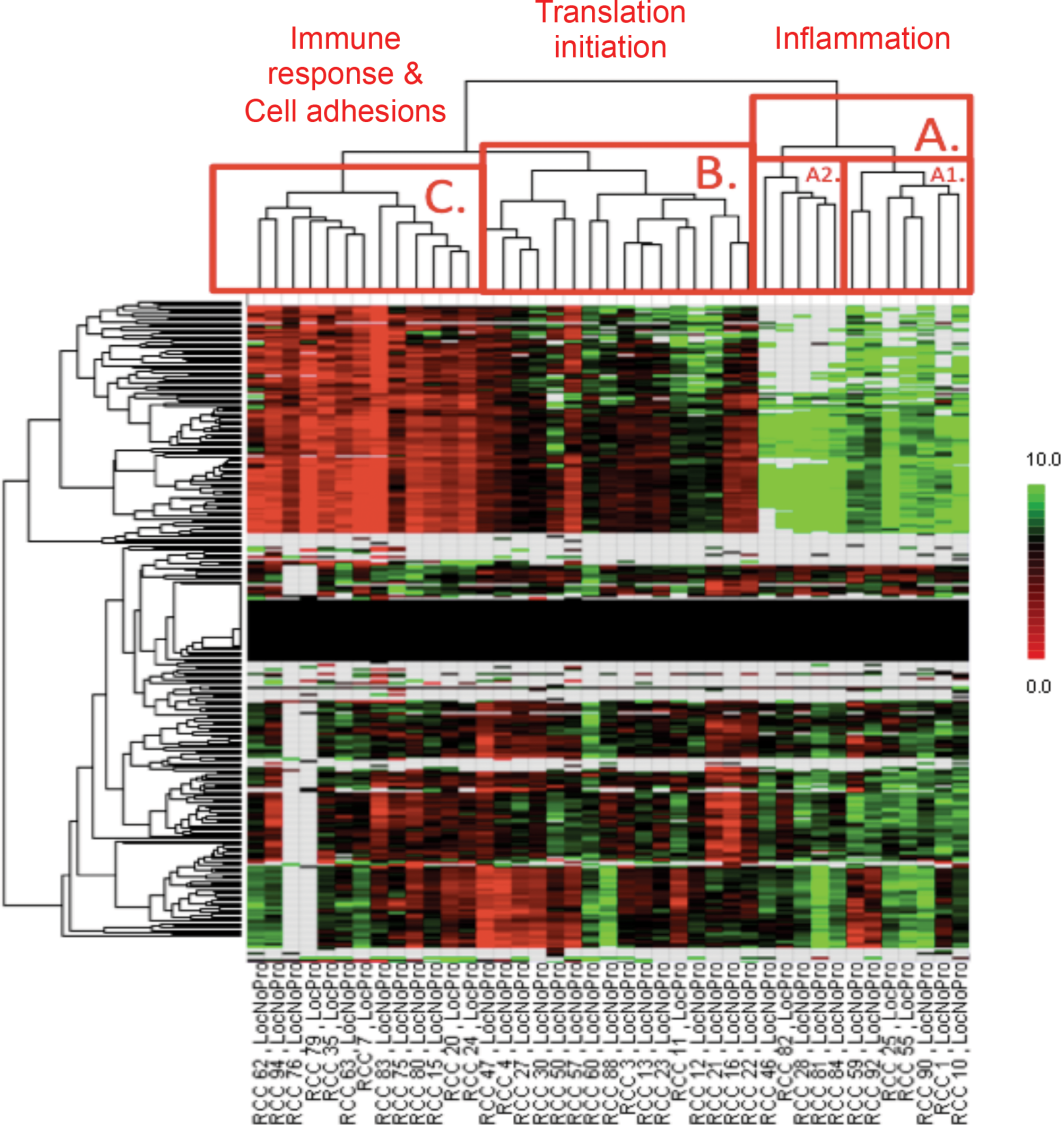
Unsupervised hierarchical clustering of kinomic profiles among localized CC-RCC. Unsupervised hierarchical clustering of kinomic peptide phosphorylation signal intensity (Y-axis) show three predominant cluster groups (labeled A, B and C on the dendrogram) among the 41 CC-RCC tumors. GeneGo MetaCore Process mapping of the significantly different peptides (p<0.002) among the clusters was performed and the dominant pathway for each cluster is indicated. Tumor number (RCC X) and clinical outcome is indicated on the X-axis (LocNoPro = locally controlled; LocPro = local progressor). Log fold changes as a deviation from the mean are displayed in the heatmap.

Kinomic analysis of the 41 localized CC-RCC tumors was performed as described in Materials and Methods (See S2 Table for raw kinomic data). To identify kinomic molecular subtypes, we used unsupervised hierarchical clustering analysis of the tumor kinomic phosphopeptide profiles to identify 3 major groups: A (N = 12), B (N = 16), and C (N = 13) (Fig 2). The phosphopeptides that defined these three groups were analyzed further using two approaches: 1) Network modeling of the protein substrates; and 2) Upstream kinase prediction.

The increased phosphopeptides for each kinomic cluster group (relative to the other groups; p<0.01) were uploaded as the parent gene Uniprot ID into GeneGo MetaCore for process mapping of the signaling networks represented in each of these 3 groups. Group specific network mappings included, but were not limited to inflammation pathways (A), translation initiation (B) and immune response and cell adhesion pathways (C) as indicated in the dendrogram of Fig 2 (Also see S1 Fig for full listing). The progression (recurrence) per kinomic cluster group is shown in Table 2. The B kinomic cluster had only 1 relapse despite being the largest subgroup (16 patients), while cluster C appeared to demonstrate the highest recurrence risk (5 of 13 recurred). Although we found a statistical association between progression and higher stage (Fisher’s Exact Test, p = 0.024), the kinomic groups were not associated with stage (Fisher’s Exact Test, p = 0.7946), suggesting that kinomic grouping is independent of stage.

**Table 2:** Relapse rates per cluster.

Cluster Group	Relapsed/Total (n)
A	25% (3/12)
B	6.3% (1/16)
C	38.5% (5/13)
Total	22% (9/41)

### Upstream kinase prediction demonstrates both tyrosine kinase-dominant and serine/threonine kinase dominant groups

**Fig 3:**
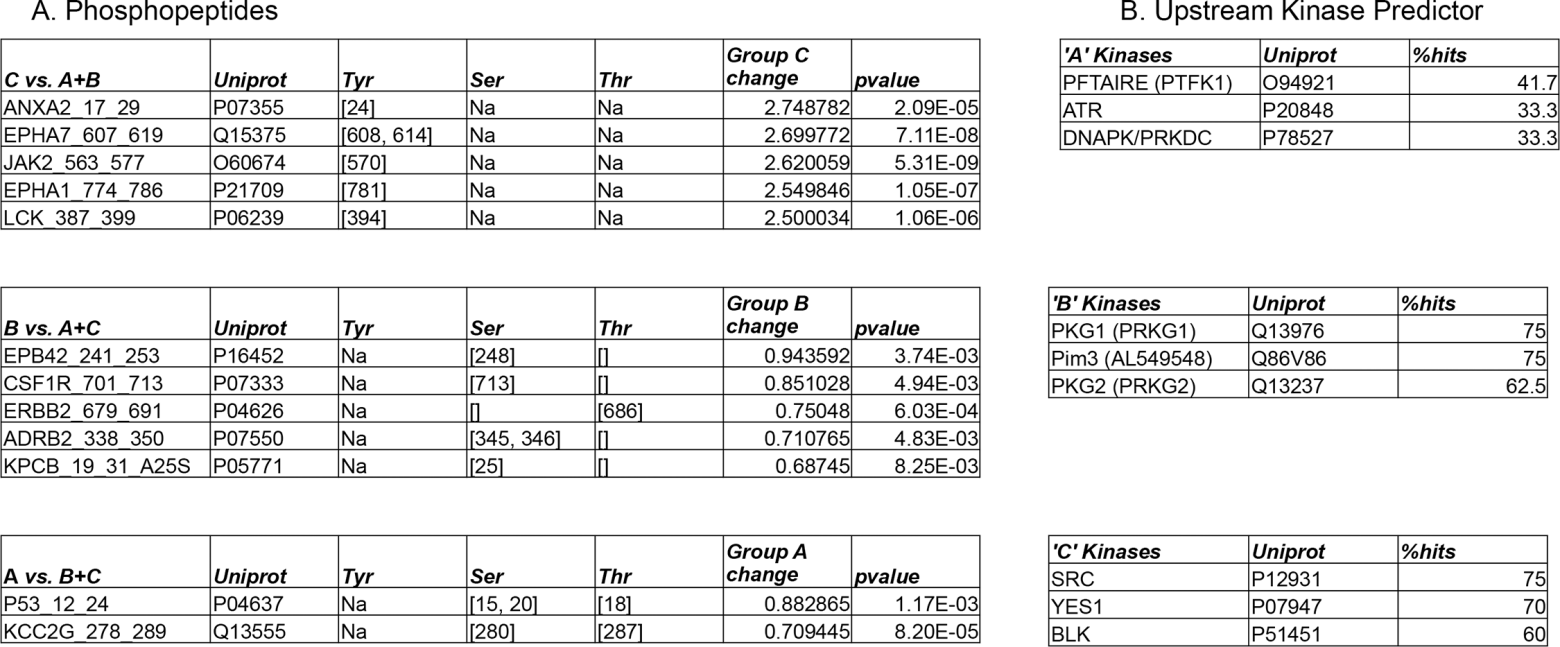
Upstream kinase prediction for kinomic cluster groups. Peptides significantly altered between cluster groups A, B and C (see Fig 2 dendrogram) are indicated in (A) and were used to query Kinexus Phosphonet to identify kinases upstream (B) of these peptides that were present in in top 10 lists for greater than 30% of those peptides (See Materials and Methods for details).

Tumors within each kinomic subgroup were compared against the other tumors, on a per-spot (per phosphopeptide) basis, using an unpaired student’s t-test (p<0.01) to identify phosphopeptides that were relatively increased in each group from Fig 2. We then utilized our upstream kinase algorithm [16] based on Kinexus kinase scoring for kinases upstream of these subtype-increased phosphopeptides. The predominant subtype-increased kinases are displayed in Fig 3 (Full listing in S2 Fig). Top potential driver kinases (per group) identified were PFTK1 (A), PKG1(B) and SRC(C).

### Kinomic comparison between CC-RCC tumors and matched adjacent normal kidney

**Fig 4:**
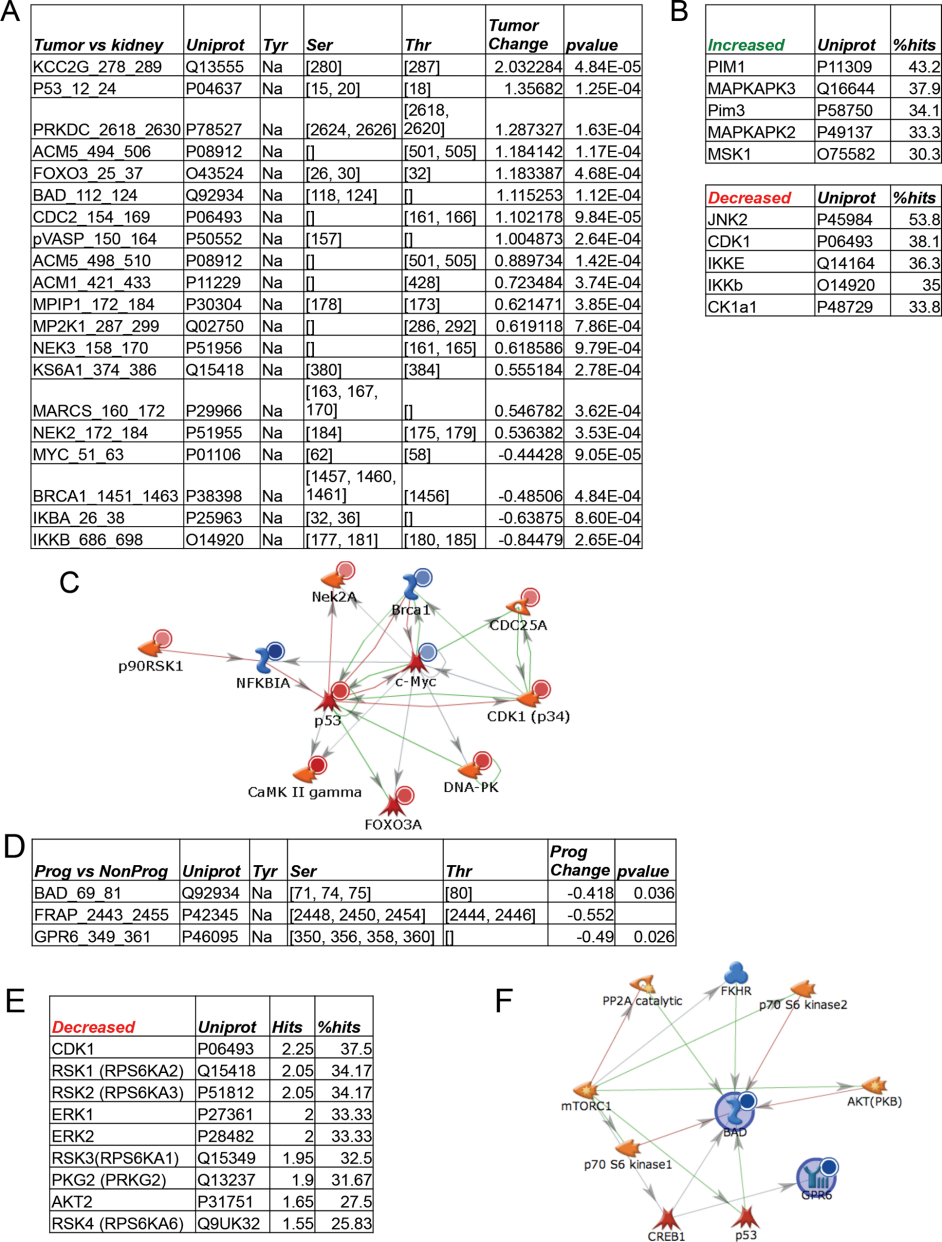
Kinases altered in CC-RCC and relationship to clinical outcome. CC-RCC tumors that had matched normal fresh frozen material available (n = 12) were directly compared and statistically different phosphopeptides (p<0.001) were identified and are shown in (A). These significant peptides were used to query Kinexus Phosphonet as in Fig 3 (and as described in Materials and Methods). Predicted upstream kinases that distinguish CC-RCC from matched normal kidney (indicated as increased or decreased in CC-RCC relative to normal kidney) are shown in (B). GeneGo MetaCore Network Modeling of the proteins that contain the significantly altered phosphopeptides (Listed as Uniprot ID’s in A) is shown in (C). Red circles indicate increased phosphorylation of the peptide while blue circles indicate decreased substrate phosphorylation. A supervised analysis of the CC-RCC tumors was performed to determine kinomic differences between patients who remained locally controlled after a minimum follow up of 18 months (NonProg) and those who progressed (Prog). Peptides significantly altered between these groups (D) were used to query Kinexus Phosphonet as above and are shown in (E) which were decreased. GeneGo MetaCore Network Modeling of the proteins containing the significantly altered phosphopeptides is shown in (F) where blue circles indicate decreased phosphorylation of the peptide.

The kinomic profiles of the 12 normal kidney tissues were directly compared with the matching CC-RCC and significantly altered phosphopeptides (p<0.001) were identified and are displayed in Fig 4A. It should be noted that the phosphopeptide changes were dominated by serine/threonine substrates (STK PamChip). Using a less stringent significance cutoff (p<0.01), 5 tyrosine substrate phosphopeptides (PTK PamChip) were significantly altered in the CC-RCC versus normal kidney (S3 Fig). Top potentially altered kinases included increased PIM’s and MAPKAPK’s and decreased JNK2 and CDK1 in tumors compared to adjacent normal renal tissue (Fig 4B). In addition, we performed network mapping of the altered phosphopeptide substrates which is shown in Fig 4C. Of note, DNA damage repair and cell cycle regulatory components were identified in this mapping.

### Supervised analysis of local progression v. locally controlled kinome profiles

We were able to perform a supervised analysis based on whether or not the patients ultimately objectively progressed. There were 9 of 41 patients that progressed (Median Time to Progression = 17 months (Range = 4–78 months). Interestingly, the statistically different phosphopeptides in the progressors versus non-progressors were predominantly serine/threonine phosphosubstrates and were reduced compared to non-progressors (Fig 4D). Upstream kinase prediction further implicated reduced CDK1 and RSK1-4 kinase activity in the progressors (Fig 4E). Network mapping of the phosphosubstrates is shown in Fig 4F. Interestingly, this map showed multiple mTOR pathway components.

## Discussion

To improve upon the limitations of current therapeutic management of CC-RCC requires a better understanding of tumor biology while also identifying and developing biomarkers for enhanced therapeutic selection. Efforts, such as those from the TCGA and others, have helped to elucidate the genomic landscape of CC-RCC.[5] CC-RCC are characterized by somatic loss of the *VHL* tumor suppressor gene in most tumors, which lead to the upregulation of HIF (Hypoxia Inducible Factor), a transcription factor that amplifies multiple proangiogenic molecules including VEGF.[18] Differential HIF-1α and HIF-2α expression has been described in *VHL*-deficient CC-RCCs, with HIF-2α expressing tumors displaying enhanced c-Myc, while those coexpressing both HIF-1α and HIF-2α or *VHL* wild type tumors displayed increased activation of Akt/mTOR and ERK/MAPK1.[19] Inactivating mutations of histone modification genes (*SETD2*, *JARID1C*, *UTX)*, a chromatin remodeling complex gene, polybromo 1 (*PBRM1)* as well as genes implicated in deubiquitination (BAP1) and the ubiquitin-mediated proteolysis pathway (UMPP) have been described.[20–23] Recently, a genetic/epigenetic study implicated the Wnt/β-catenin pathway in renal cell carcinogenesis.[24] Baseline molecular profiling of tumors may also improve prediction of recurrence, e.g. single nucleotide polymorphisms (SNPs) of MET, and response to sunitinib, e.g. SNPs in VEGFR3 and CYP3A5*1.[25, 26]

Our present study complements these efforts by providing the first published comprehensive kinomic evaluation of localized CC-RCC. In our samples, despite the wide range of clinical stages, we identified three distinct kinomic clusters among these primary tumors using an unsupervised classification strategy (hierarchical clustering). Because there are multiple statistical approaches for clustering microarray data, it is important to determine the robustness of dendrogram classification as small changes in data points included can potentially change the clustering of the samples. Thus, we performed a secondary hierarchical clustering strategy with bootstrap resampling to address our classification quality (pvclust R script). Although some filtering of the raw data was required to do this (e.g. removing very low signal strength values), in general, we found that the samples continued to cluster in the identified groups. Moreover, many of the dendrogram branches had very high AU scores indicating that the data highly supported the clustering (See S4 Fig). Importantly, our bioinformatic approach elucidated distinguishing biological processes and enriched for dominant kinases within each cluster. Moreover, one of the clusters was associated with excellent prognosis (Cluster Group B–Translation-initiation process dominant) while one had the preponderance of recurrences (Cluster Group C–Immune Response and Cell Adhesion process dominant). This “basal” or “inherent” kinomic profile information not only gives insight regarding potential kinase-driven (and, thus, targetable) pathways in CC-RCC but also is suggestive of prognostic utility.

Interestingly, the heretofore clinically validated kinase targets, VEGF receptors and kinases in the mTOR signaling pathway did not emerge as the top ranked active kinases. While preliminary data indicate that inhibition of MET in addition to VEGF receptor kinase inhibition may yield clinical benefits (with cabozantinib), this kinase was also not among the top altered kinases in our unsupervised or supervised analyses.[10] Nevertheless, when comparing progressors vs. non-progressors, signaling kinases downstream of these key kinases did emerge as potentially important drivers of disease as is illustrated by increased SRC kinase activity in group C of the unsupervised analysis, increased PIM’s and MAPKAPK’s in tumors compared to adjacent matched normal, and reduced CDK1 and RSK1-4 kinase activity with altered mTOR components. The depressed activities of multiple kinases in progressors compared to non-progressors suggest that not all kinase inhibition is beneficial and even that induction of certain kinases with tumor inhibiting activity may be a strategy worth exploring.[27] Indeed, the silencing of tumor suppressor genes appears more prevalent than activated oncogenes in CC-RCC and most other malignancies. However, the kinomic platform employed in our study measures kinase activity and not the quantity of kinases. Thus, the activity of kinases that are frequently modulated by post-translational modification may not correlate linearly with kinase protein levels. It is also notable that most other platforms assaying at the DNA and RNA levels may be quantitative measures for specific alterations (e.g. mutations, copy numbers) but do not measure how those alterations affect kinase functional activity.

Our study is limited by its small sample size and retrospective design. For our analyses, we chose to focus on 41 patients with localized CC-RCC undergoing definitive surgery for whom we had satisfactory follow-up defined as ≥18 months for non-progressors. Thus, despite the small sample size, all patients harbored pathology verified CC-RCC that were clinically well-annotated and the analysis utilized objective tumor recurrence as the primary clinical endpoint and not overall survival or progression-free survival, which are confounded by non-cancer mortalities. Indeed, stage was statistically associated with tumor recurrence, which improves the confidence in our tumor tissue kinomics data. As suspected, tumor biology was not concordant with stage, since kinomic profiles were not associated with stage. We acknowledge that the substantial intra- and inter-patient genomic tumor heterogeneity remains a formidable challenge when attempting to identify broad deterministic patterns as opposed to stochastic variations.[28, 29] Unfortunately, we did not have data regarding outcomes with therapy for recurrent tumors, and hence could not correlate the activity of therapy with kinomics. Finally, pre-analytic variables such as time from renal vessel clamping to nephrectomy to tumor harvesting may impact kinase activity and were not taken into account. However, previous analyses have found marginal impact for pre-analytic variability.[30] It should also be noted that due to tissue and cost limitations, the samples were run as singlicates but statistically analyzed as “biological-replicates” based on progression status. We felt justified in this approach based on the high technical reproducibility of the PamStation (based on coefficient of variance) using only 2–10 μg of protein lysate derived from a small quantity of viable tissue.[27, 31–33]

To conclude, this is the first study to propose a classification scheme for CC-RCC based on activity of kinases in tumor tissue. The study identifies 3 distinct kinomics patterns with different dominant pathways active in these subtypes. Also, differences emerged when comparing tumors with adjacent normal renal tissue and progressors vs. non-progressors. Our data warrant external validation in a larger clinically annotated dataset, and optimally, prospective validation obeying REMARK guidelines for prognostic biomarker validation.[34] Moreover, activity of commonly employed TKIs, pazopanib and sunitinib, upon metastatic recurrence, could be correlated with baseline tumor tissue kinomics in order to discover panels predictive for benefit. Interestingly, the kinomic profile demonstrating the highest recurrence rates following surgery exhibited activation of immune response, suggesting a potential role for the emerging highly promising Programmed Death (PD)-1 inhibitors as an adjuvant strategy for patients with tumors exhibiting certain kinomic profiles.[35]

## Supporting Information

S1 Table Detailed patient characteristics. (PDF)

S2 Table Complete kinomic data from 41 localized CC-RCC tumors. This file contains the raw data by cycle number as well as log2 transformed data. NA indicates that data point was censored due to not passing quality control screening for signal strength in BioNavigator.
(XLSX)

S1 Fig Process pathway maps from GeneGo MetaCore. The unsupervised hierarchical clustering of kinomic peptide phosphorylation signal intensity identified three predominant cluster groups (labeled A, B and C on the dendrogram) among the 41 CC-RCC tumors as shown in Fig 2. GeneGo MetaCore Process mapping of the significantly different peptides (p<0.002) among the clusters was performed and the full listing is shown here.
(PDF)

S2 Fig Upstream kinase prediction for kinomic cluster groups. Peptides significantly altered between cluster groups A, B and C (see Fig 2 dendrogram) from Fig 3A were used to query Kinexus Phosphonet to identify kinases upstream of these peptides that were present in in top 10 lists for greater than 30% of those peptides. The full listing is shown here.
(PDF)

S3 Fig Tyrosine Kinases altered in CC-RCC. CC-RCC tumors that had matched normal fresh frozen material available (n = 12) were directly compared and statistically different phosphopeptides (p<0.01) were identified from the PTK PamChip and are shown in (A). These significant peptides were used to query Kinexus Phosphonet as in Fig 4. Predicted upstream tyrosine kinases that distinguish CC-RCC from matched normal kidney (indicated as increased in CC-RCC relative to normal kidney) are shown in (B). Of note, the tyrosine kinases were scored for presence within the 3 increased peptides (*AMPE_5_17 did not have a kinexus entry). Kinases (AXL and EGFR both scoring 4/6 or 66%) were excluded if they appeared upstream in the single decreased kinase list (PLCG1_1246_1258). GeneGo MetaCore Network Modeling of the proteins that contain the significantly altered phosphopeptides (Listed as Uniprot ID’s in A) is shown in (C). Red circles indicate increased phosphorylation of the peptide while blue circles indicate decreased substrate phosphorylation.
(PDF)

S4 Fig Alternate hierarchical clustering with bootstrap resampling p-value calculations. For validation of Heatmap Clustering (Fig 2) complete kinomic peptide profiles were clustered using the R script *pvclust* with average euclidean distances to generate Approximately Unbiased (AU) probability values (red text in dendrogram) and Bootstrap Probabilities (100 bootstrap replications; green text in dendrogram) to test the robustness of the clustering (Suzuki and Shimodaira Bioinformatics 2006). All peptides that had NA or zero values in any replicate were removed as per script requirements. Samples that clustered with an AU greater than 95 AU, are boxed in red (generated using R version 2.15.3 (r-project.org) and Rstudio version 0.98.1103).
(PDF)
