# Supplementary material for: High Throughput Kinomic Profiling of Human Clear Cell Renal Cell Carcinoma Identifies Kinase Activity Dependent Molecular Subtypes
Source: PLoS One. 2015 Sep 25;10(9):e0139267. doi: 10.1371/journal.pone.0139267 (PMC4583516; doi:10.1371/journal.pone.0139267)
Supplement: S1 Table — (PDF) [file pone.0139267.s001.pdf]

Supporting Table 1. Detailed patient characteristics

ID	Age at Diagnosis	Gender (1=M, 0=F)	TNM	Stage at diagnosis	Progressed (0=no, 1=yes)	% tumor	% necrosis
RCC 1	62	1	T3bN0M0	3	0	10	0
RCC 10	84	1	T1aN0M0	1	0	<10	0
RCC 11	55	0	T3bN0M0	3	1	100	80
RCC 12	33	1	T1N0M0	1	0	60	20
RCC 13	72	0	T3aN0M0	4	0	100	30
RCC 15	73	1	T1N0M0	1	0	20	10
RCC 16	69	0	T1N0M0	1	0	40	0
RCC 20	77	1	T3aN2M0	3	1	90	10
RCC 21	61	1	T1N0M0	1	0	100	20
RCC 22	56	1	T1aN0M0	1	0	90	10
RCC 23	54	1	T3aN0M0	3	0	100	10
RCC 24	85	1	T1aN0M0	3	1	80	10
RCC 25	66	1	T3aN0M0	3	1	80	70
RCC 27	46	0	T1aN0M0	3	0	90	50
RCC 28	55	1	T1bN0M0	1	0	30	50
RCC 3	78	1	T1aN0M0	3	0	70	0
RCC 30	60	1	T1bN0M0	1	0	100	10
RCC 35	67	1	T1N0M0	1	1		
RCC 4	54	1	T3aN0M0	3	0	80	0
RCC 46	47	1	T1N0M0	1	0	60	20
RCC 47	64	1	T1aN0M0	1	0	100	10
RCC 50	47	0	unavailable	1	0		
RCC 55	68	1	T3aN0M0	3	1	70	50
RCC 57	68	1	T1bN0M0	1	0	80	0
RCC 59	71	1	T3N0M0	3	0	100	50
RCC 60	53	0	T3bN0M0	3	0	10	0
RCC 62	59	1	T1N0MO	1	0	100	10
RCC 63	62	1	T3N0M0	3	0	100	70
RCC 7	56	1	T3bN0M0	3	1	100	20
RCC 75	54	0	T2aN0M0	2	0	100	20
RCC 76	51	0	T1N0M0	1	0	90	10
RCC 79	61	1	T3bN0M0	3	1	100	60
RCC 80	67	1	T2N0M0	3	0	100	50
RCC 81	65	0	T1bN1M0	3	0	30	20
RCC 82	56	1	T3bN0M0	4	1	90	20
RCC 83	37	0	T1N0M0	1	0	70	20
RCC 84	60	1	T1aN0M0	3	0	100	20
RCC 88	61	1	unavailable	1	0		
RCC 90	67	0	unavailable	1	0		
RCC 92	57	0	unavailable	1	0		
RCC 94	64	1	unavailable	1	0		
