# Supplementary material for: High Throughput Kinomic Profiling of Human Clear Cell Renal Cell Carcinoma Identifies Kinase Activity Dependent Molecular Subtypes
Source: PLoS One. 2015 Sep 25;10(9):e0139267. doi: 10.1371/journal.pone.0139267 (PMC4583516; doi:10.1371/journal.pone.0139267)
Supplement: S1 Fig — The unsupervised hierarchical clustering of kinomic peptide phosphorylation signal intensity identified three predominant cluster groups (labeled A, B and C on the dendrogram) among the 41 CC-RCC tumors as shown in Fig 2. GeneGo MetaCore Process mapping of the significantly different peptides (p<0.002) among the clusters was performed and the full listing is shown here. (PDF) [file pone.0139267.s003.pdf]

Supplemental Figure S1

Top Ranked 'A' Processes	Top Ranked 'B' Processes	Top Ranked 'C' Processes
Inflammation_MIF signaling	Reproduction_GnRH signaling pathway	Immune response_Phagocytosis
Inflammation_Amphoterin signaling	Reproduction_Gonadotropin regulation	Apoptosis_Anti-Apoptosis mediated by external signals via PI3K/AKT
DNA damage_Checkpoint	Neurophysiological process_Long-term potentiation	Immune response_Phagosome in antigen presentation
Cell cycle_G1-S Interleukin regulation	Signal Transduction_Cholecystokinin signaling	Cell adhesion_Platelet aggregation
Development_Hemopoiesis, Erythropoietin pathway	Translation_Regulation of initiation	Cell adhesion_Glycoconjugates
Inflammation_IgE signaling	Development_Melanocyte development and pigmentation	Inflammation_NK cell cytotoxicity
Immune response_BCR pathway	Inflammation_Histamine signaling	Apoptosis_Anti-Apoptosis mediated by external signals via MAPK and JAK/STAT
Inflammation_TREM1 signaling	Neurophysiological process_Transmission of nerve impulse	Cell adhesion_Leucocyte chemotaxis
Development_Neuromuscular junction	Neurophysiological process_Circadian rhythm	Development_Hemopoiesis, Erythropoietin pathway
Immune response_TCR signaling	Muscle contraction_Relaxin signaling	Immune response_BCR pathway
