# Supplementary material for: High Throughput Kinomic Profiling of Human Clear Cell Renal Cell Carcinoma Identifies Kinase Activity Dependent Molecular Subtypes
Source: PLoS One. 2015 Sep 25;10(9):e0139267. doi: 10.1371/journal.pone.0139267 (PMC4583516; doi:10.1371/journal.pone.0139267)
Supplement: S2 Fig — Peptides significantly altered between cluster groups A, B and C (see Fig 2 dendrogram) from Fig 3A were used to query Kinexus Phosphonet to identify kinases upstream of these peptides that were present in in top 10 lists for greater than 30% of those peptides. The full listing is shown here. (PDF) [file pone.0139267.s004.pdf]

Supplemental Figure S2

'A' Kinases	Uniprot	Hits	Score	%hits
PFTAIRE1 (PFTK1)	O94921	1.6665	5201.147	41.6625
ATR	Q13535	1.3332	5111.489	33.33
DNAPK/PRKDC	P78527	1.3332	4327.567	33.33
JNK1 (MAPK8)	P45983	1.3332	4275.572	33.33
JNK3 (MAPK10)	P53779	1.3332	4275.572	33.33
ERK1	P27361	1.3332	4031.597	33.33
ERK2 (MAPK1)	P28482	1.3332	3988.934	33.33
CDK1 (CDC2)	P06493	1.3332	3964.937	33.33
JNK2 (MAPK9)	P45984	1.3332	3659.634	33.33

'B' Kinases	Uniprot	Hits	Score	%hits
PKG1 (PRKG1)	Q13976	6	20400	75
Pim3 (AL549548)	P58750	6	14316	75
PKG2 (PRKG2)	Q13237	5	15225	62.5
PKACa (PRKACA)	P17612	5	11950	62.5
MSK1 (RPS6KA5)	O75582	4	10264	50
p70S6Kb (RPS6KB2)	Q9UBS0	4	7468	50
PRKX	P51817	3	5730	37.5
PKACb PRKACB	P22694	3	5658	37.5

'C' Kinases	Uniprot	Hits	Score	%hits
SRC	P12931	7.5	60660	75
YES1	P07947	7	56280	70
BLK	P51451	6	36360	60
PYK2 (PTK2B)	Q14289	5	34950	50
HCK	P08631	5	34860	50
FYN	P06241	5	27120	50
SYK	P43405	5	25265	50
ERBB3	P21860	4.5	14350.5	45
ARG (ABL2)	P42684	4	17096	40
LYN	P07948	4	16004	40
CTK (MATK)	P42679	4	9676	40
BRK	Q13882	3	8700	30
AXL	P30530	3	7479	30
LCK	P06239	3	7323	30
FGR	P09769	3	6327	30
