# Supplementary material for: High Throughput Kinomic Profiling of Human Clear Cell Renal Cell Carcinoma Identifies Kinase Activity Dependent Molecular Subtypes
Source: PLoS One. 2015 Sep 25;10(9):e0139267. doi: 10.1371/journal.pone.0139267 (PMC4583516; doi:10.1371/journal.pone.0139267)

Supplemental Figure S3

A

PTK Peptides	Change with Tumor
AMPE_5_17 *	0.186
DDR1_506_518	2.135
EPHB1_921_933	0.621
NPT2A_501_513	0.25
PLCG1_1246_1258	-0.522

B

Kinase	Uniprot	Hits	% Hits
ERBB2	P04626	6	100
ERBB4	Q15303	5	83.33
MER (MERTK)	Q12866	5	83.33
ABL	P00519	3	50

C

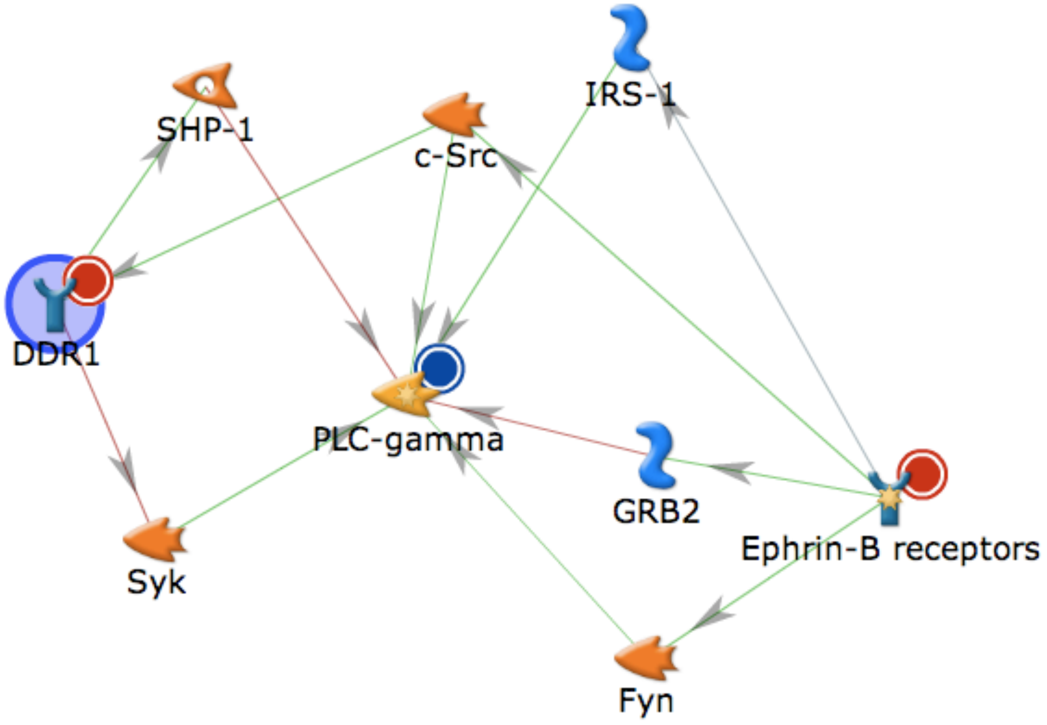

Supplement: S3 Fig — CC-RCC tumors that had matched normal fresh frozen material available (n = 12) were directly compared and statistically different phosphopeptides (p<0.01) were identified from the PTK PamChip and are shown in (A). These significant peptides were used to query Kinexus Phosphonet as in Fig 4. Predicted upstream tyrosine kinases that distinguish CC-RCC from matched normal kidney (indicated as increased in CC-RCC relative to normal kidney) are shown in (B). Of note, the tyrosine kinases were scored for presence within the 3 increased peptides (*AMPE_5_17 did not have a kinexus entry). Kinases (AXL and EGFR both scoring 4/6 or 66%) were excluded if they appeared upstream in the single decreased kinase list (PLCG1_1246_1258). GeneGo MetaCore Network Modeling of the proteins that contain the significantly altered phosphopeptides (Listed as Uniprot ID’s in A) is shown in (C). Red circles indicate increased phosphorylation of the peptide while blue circles indicate decreased substrate phosphorylation. (PDF) [file pone.0139267.s005.pdf]
