# Supplementary figures and images for: High Throughput Kinomic Profiling of Human Clear Cell Renal Cell Carcinoma Identifies Kinase Activity Dependent Molecular Subtypes
Source: PLoS One. 2015 Sep 25;10(9):e0139267. doi: 10.1371/journal.pone.0139267 (PMC4583516; doi:10.1371/journal.pone.0139267)

# Supporting Figure S4

Cluster Dendrogram with AU/BP Values

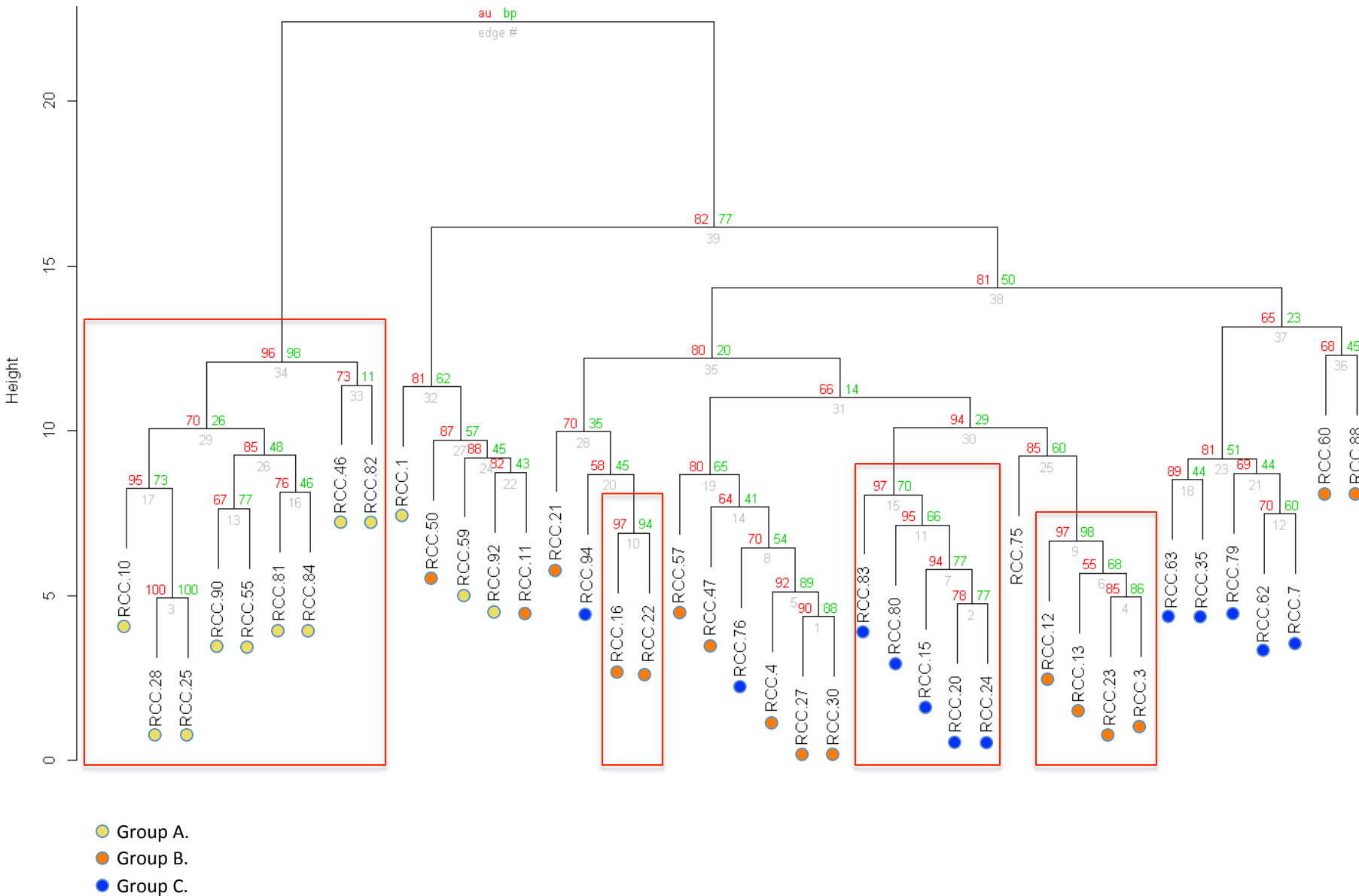

Supplement: S4 Fig — For validation of Heatmap Clustering (Fig 2) complete kinomic peptide profiles were clustered using the R script pvclust with average euclidean distances to generate Approximately Unbiased (AU) probability values (red text in dendrogram) and Bootstrap Probabilities (100 bootstrap replications; green text in dendrogram) to test the robustness of the clustering (Suzuki and Shimodaira Bioinformatics 2006). All peptides that had NA or zero values in any replicate were removed as per script requirements. Samples that clustered with an AU greater than 95 AU, are boxed in red (generated using R version 2.15.3 (r-project.org) and Rstudio version 0.98.1103). (PDF) [file pone.0139267.s006.pdf]
